# Design and Delivery Features That May Improve the Use of Internet-Based Cognitive Behavioral Therapy for Children and Adolescents With Anxiety: A Realist Literature Synthesis With a Persuasive Systems Design Perspective

**DOI:** 10.2196/11128

**Published:** 2019-02-05

**Authors:** Ashley D Radomski, Lori Wozney, Patrick McGrath, Anna Huguet, Lisa Hartling, Michele P Dyson, Kathryn Bennett, Amanda S Newton

**Affiliations:** 1 Department of Pediatrics University of Alberta Edmonton, AB Canada; 2 Centre for Research in Family Health IWK Health Centre Halifax, NS Canada; 3 Department of Psychology Dalhousie University Halifax, NS Canada; 4 Department of Pediatrics Dalhousie University Halifax, NS Canada; 5 Deparment of Psychiatry Dalhousie University Halifax, NS Canada; 6 Department of Community of Health and Epidemiology Dalhousie University Halifax, NS Canada; 7 Department of Health Research Methods, Evidence and Impact McMaster University Hamilton, ON Canada

**Keywords:** internet, cognitive behavioral therapy, computer-assisted therapy, persuasive communication, anxiety, children, adolescents, review, adherence

## Abstract

**Background:**

Internet-based cognitive behavioral therapy (iCBT) is a *persuasive system* as its design combines therapeutic content, technological features, and interactions between the user and the program to reduce anxiety for children and adolescents. How iCBT is designed and delivered differs across programs. Although iCBT is considered an effective approach for treating child and adolescent anxiety, rates of program use (eg, module completion) are highly variable for reasons that are not clear. As the extent to which users complete a program can impact anxiety outcomes, understanding what iCBT design and delivery features improve program use is critical for optimizing treatment effects.

**Objective:**

The objectives of this study were to use a realist synthesis approach to explore the design and delivery features of iCBT for children and adolescents with anxiety as described in the literature and to examine their relationship to program use outcomes.

**Methods:**

A search of published and gray literature was conducted up to November 2017. Prespecified inclusion criteria identified research studies, study protocols, and program websites on iCBT for child and adolescent anxiety. Literature was critically appraised for relevance and methodological rigor. The persuasive systems design (PSD) model, a comprehensive framework for designing and evaluating persuasive systems, was used to guide data extraction. iCBT program features were grouped under 4 PSD categories—Primary task support, Dialogue support, System credibility support, and Social support. iCBT design (PSD Mechanisms) and delivery features (Context of use) were linked to program use (Outcomes) using meta-ethnographic methods; these relationships were described as Context-Mechanism-Outcome configurations. For our configurations, we identified key PSD features and delivery contexts that generated moderate-to-high program use based on moderate-to-high quality evidence found across multiple iCBT programs.

**Results:**

A total of 44 documents detailing 10 iCBT programs were included. Seven iCBT programs had at least one document that scored high for relevance; most studies were of moderate-to-high methodological rigor. We developed 5 configurations that highlighted 8 PSD features (Tailoring, Personalization [Primary task supports]; Rewards, Reminders, Social role [Dialogue supports]; and Trustworthiness, Expertise, Authority [System credibility supports]) associated with moderate-to-high program use. Important features of delivery Context were adjunct support (a face-to-face, Web- or email-based communications component) and whether programs targeted the prevention or treatment of anxiety. Incorporating multiple PSD features may have additive or synergistic effects on program use.

**Conclusions:**

The Context-Mechanism-Outcome configurations we developed suggest that, when delivered with adjunct support, certain PSD features contribute to moderate-to-high use of iCBT prevention and treatment programs for children and adolescents with anxiety. Standardization of the definition and measurement of program use, formal testing of individual and combined PSD features, and use of real-world design and testing methods are important next steps to improving how we develop and deliver increasingly useful treatments to target users.

## Introduction

### Background

Cognitive behavioral therapy (CBT) is recommended as a first-line treatment for children and adolescents with anxiety [1-4]. Trained mental health professionals have traditionally delivered CBT, but there is an increasing interest in the internet as a delivery platform to circumvent the multiple barriers to receiving in-person treatment. These barriers can include direct and incidental costs to families, lack of trained deliverers, and inconvenient service times and locations [5]. Internet-based CBT (iCBT) is also proposed to preserve adolescent autonomy, appeal to user preferences, reduce health care system costs, and improve the time it takes to receive treatment [6-8]. iCBT is recognized as an important treatment option [9-11] to meet the increasing demands of a population where anxiety is often undetected and untreated, but with whom early access to care can improve long-term outcomes [3,12-14].

iCBT uses technological features (ie, multimedia and email) to deliver treatment content through the Web or a software application to generate interactions between the user and the program [15]. In this way, iCBT aims to reinforce, change, or shape attitudinal or behavioral health outcomes and aligns with the concept of a *persuasive system* [16-18]. Although recent efforts have been made to provide guidance on the design and delivery features of iCBT [19], considerable differences exist across programs both in terms of their features and the health outcomes they produce. Recent systematic reviews and meta-analyses have found a range of iCBT programs to be effective at reducing anxiety in children and adolescents [9,10,20,21]; however, poor and highly variable rates of completion can be found across programs (up to 50% of participants not reaching the end of a program) [5,7,10,21,22]. The term *program use* captures the various, typically objective, outcomes used across studies (ie, adherence, compliance, and number of program activities or homework completed) that describe the extent to which users interact with a program.

Understanding the factors that influence iCBT program use is important as there have been indications that greater program use is associated with better outcomes [23,24]. Studies of iCBT in children and adolescents with anxiety [25,26] have found that certain participant demographics (eg, gender, age, location, and anxiety severity) and delivery features (eg, parental or therapist support) relate to or predict program use. However, the relationship between technological design features of a program, the ability of those features to enhance the *persuasiveness* of a program, and actual iCBT program use by children and adolescents with anxiety, has received minimal attention in the literature.

### Objectives

Recognizing iCBT as a persuasive system, we conducted a realist synthesis to examine the technological design and program delivery features of iCBT for children and adolescents with anxiety in order to document their potential relation to persuading program use. The realist synthesis approach provided a framework to answer 2 main questions: (1) what design and delivery components (technological features, treatment content, and interactions) are described for iCBT programs for children and adolescents with anxiety? and (2) what components may explain reported program use outcomes?

## Methods

### Study Design

This realist synthesis was conducted using steps recommended by Pawson and Tilley [27,28] and is reported in accordance with the Realist and Meta-narrative Evidence Synthesis: Evolving Standards (RAMESES II) [29]. Realist synthesis is theory-driven in that the synthesis searches for and refines explanations of intervention effects by asking: “What works, for whom, and in what circumstances?” [27]. In this synthesis, we examined the relationships that exist between the therapeutic and technological features of iCBT (Mechanisms) and program use (Outcomes), and the program delivery formats and interactions (Contexts) that support them. We expressed these relationships as Context-Mechanism-Outcome configurations.

### Context-Mechanism-Outcome Configuration Development

We began the synthesis by developing *candidate* Context-Mechanism-Outcome configurations for how iCBT programs may work. The development process consisted of brainstorming activities, with the research team reviewing literature on human-technology interaction and studies of iCBT programs for anxiety to identify relevant and pre-existing theories, models, or frameworks to work from. The persuasive systems design (PSD) model [17] emerged as a key framework for understanding how iCBT, as a persuasive system, was intended to work, and we used this model to develop the initial list of Context-Mechanism-Outcome configurations. The model describes 28 PSD (technological) features, subdivided across the following 4 categories, which can be implemented by programs to guide the user toward their health-related goal: (1) Primary task support, (2) Dialogue support, (3) System credibility support, and (4) Social support.

Using the PSD model, we identified which PSD features (Mechanisms) might be associated with iCBT program use (Outcomes) to formulate Mechanism-Outcome dyads. We then hypothesized which program delivery formats, interactions, and conditions for use (Context) might promote the occurrence of the Mechanism-Outcome interactions. Together, these steps led to the generation of 5 candidate Context-Mechanism-Outcome configurations (Multimedia Appendix 1) [30-34]. The configurations were as comprehensive and justifiable as possible, referencing literature that supported their development and inclusion in the list. These configurations would undergo refinement and testing during the analysis stage of the synthesis, whereby we used evidence from the literature to validate their explanatory usefulness and applicability for answering our research questions.

### Literature Search

We used 3 main strategies to identify literature for iCBT programs. The first search strategy involved an information specialist conducting a systematic and comprehensive search of 8 electronic databases: MEDLINE, EMBASE, ERIC, PsycINFO, CINAHL, Cochrane Library, ProQuest Dissertations & Theses Global, and PubMed for the period 1990 to 2017. The second strategy involved a manual search using Google, an internet search engine, and gray literature repositories (Association for Computing Machinery Digital Library, Open Grey, Institute of Electrical and Electronics Engineers Digital Library, and Canadian Agency for Drugs and Technologies in Health) to identify conference proceedings, program evaluations, and government or technical reports. For both search strategies, MeSH terms and text words were based on mental health condition (anxiety and phobias), intervention modality (internet-based and mobile app), intervention type (prevention and treatment), and therapeutic approach (CBT; Multimedia Appendix 2). The third strategy involved manually searching the table of contents in the *Journal of Medical Internet Research*, *Internet Interventions*, *Journal of Cyber Therapy & Rehabilitation*, and *Journal of Telemedicine and Telecare*, and a review of reference lists of included documents and reviews (eg, systematic reviews).

We employed the search strategies on an iterative and recurrent basis until November 2017 to ensure the review was up to date and inclusive. Before discontinuing the literature search, a test of saturation was applied to the search strategies, which involved verifying that further searching would not yield any new results [35].

### Literature Selection

In this study, 2 independent, trained reviewers (authors ADR and LW) screened the identified documents for eligibility using a 2-stage approach. During this process, reviewer discrepancies were resolved by consensus or third party arbitration (author ASN). At stage 1, all documents were screened for eligibility using the title and abstract. During this stage, we randomly selected 100 citations to assess inter-rater agreement for inclusion or exclusion decisions; Cohen kappa was 0.74 between raters, reflecting substantial agreement [36]. All documents that were screened “yes, include” or “unsure to include” moved to stage 2. At stage 2, the full text of documents was reviewed by 1 reviewer (ADR), in consultation with another (ASN), with a resulting decision to either include or exclude a document from the synthesis.

For an iCBT program to be included in this synthesis, supporting documents needed to be published in English and provide information on treatment Context, program design (PSD) and delivery features, and program use Outcomes. Each document did not need to provide details on all 3, but all 3 needed to be represented in the total documents for an iCBT program. In addition, at least one published study on the iCBT program needed to be available for inclusion so that we could assess the methodological quality of the study providing program use outcome data.

Intervention studies (eg, clinical trials) were eligible for inclusion if they evaluated iCBT anxiety programs with children (aged <14 years) or adolescents (aged 12-19 years). As some iCBT programs were designed for and evaluated with participants from a broader age range (eg, programs also geared toward young adults), only those studies that provided separate data for participants aged ≤19 years were included. We also required that the type of iCBT program under evaluation be designed for an anxiety disorder(s) or anxiety symptoms associated with a disorder as classified according to the Diagnostic and Statistical Manual of Mental Disorders, fifth edition [37] such as social phobia (social anxiety disorder), generalized anxiety disorder, panic disorder, separation anxiety disorder, or specific phobia. Transdiagnostic programs (ie, programs designed for anxiety plus another diagnosis such as depression) were also eligible for inclusion. We also required that the iCBT program consisted of modules designed for use by the child or adolescent (and not solely delivered to or facilitated by a parent or therapist) as child or adolescent program use was our outcome of interest. Theoretical papers, mixed-methods and qualitative studies, and policy or implementation documents were also eligible if they included a focus on how an iCBT program was proposed to work.

### Literature Appraisal

Documents were assessed for relevance and rigor based on consensus between 2 reviewers (authors ADR and LW). Relevance was assessed based on the level of contribution a document provided for an iCBT program in 3 domains: (1) underpinning theory and/or the context and sequence for delivery (Context), (2) PSD features (Mechanism), and (3) program use outcomes (Outcomes). The level of contribution for each domain was rated *low* if little or no information was provided, *medium* if some information was provided, and *high* if information was well described. Exemplar documents with a *high* level of contribution across the 3 domains informed decision rules for the rating of all other documents.

The methodological quality (rigor) of research studies was assessed using the Mixed Methods Appraisal Tool (MMAT) [38,39]. The MMAT is a reliable, efficient, and valid tool that provides different sections for assessing studies of qualitative, randomized, nonrandomized studies, descriptive studies, and mixed-method designs [38-40]. Multiple publications of the same study (thesis + journal publication) received the same MMAT score. MMAT scores could range from 0% to 100%, with a greater score indicating that more quality criteria were met.

### Data Extraction and Coding

Documents for each iCBT program were grouped together during data extraction and coding. Two reviewers (ADR and LW) cross-referenced extraction and coding decisions, with a random subset of 10 documents; the remaining documents were coded by 1 reviewer (ADR). In addition to document characteristics (type and year of publication), iCBT program data were extracted for the following:

- Participants: eligibility criteria and participant demographics.
- iCBT program Context: number of modules, module workflow and sequence, delivery setting, adjunct support, and level of prevention according to the Institute of Medicine model [41].
- Theory or proposed Mechanisms: program features, content and components, including PSD features, and information on why the iCBT program was designed a certain way or how the program was proposed to work.
- Program use Outcomes: information related to how many Web-based modules, exercises, or activities were completed by users or how many users completed certain aspects of the program, measured at posttreatment.

#### Context and Mechanism Data

Adjunct support details were coded when human-derived technological or therapeutic communication was provided to users to complement iCBT program delivery.

Therapeutic content in programs was coded according to the 5 main CBT components found in the American Academy of Child and Adolescent Psychiatry (AACAP) practice parameter [42]: psychoeducation, somatic management skills, cognitive restructuring, exposure methods, and relapse prevention.

As most authors did not use PSD terminology or concepts, program descriptions were coded as PSD features using a codebook and glossary [17] (Multimedia Appendix 3). PSD features were coded when they were executed by the technology (intrinsic to the design and delivery of the internet-based program) and not by human action (eg, congratulatory comments provided in person by a parent or teacher), which is in line with the use of the PSD model by others [15]. When available, suggested mediators and moderators of program use were extracted, as was information on partial or full Context-Mechanism-Outcome configurations, as discussed by the original authors of the included documents.

#### Outcome Data

We found inconsistent and heterogeneous measurement and reporting of program use Outcomes—such as program adherence, compliance, and completion. These limitations have been noted by others [22].

For each study, program use Outcomes are reported as published by original authors and are collectively referred to in this study using the umbrella term *program use*. As no applicable cut-off scores have yet been established, when possible, Outcomes were converted into percentages (based on quartiles) to assist with interpreting program use. We used the following parameters to summarize program use: *high* use (≥75%), *moderate* use (50%-74%), *low* use (25%-49%), or *very low* use (≤24%). Study dropout or attrition data were not included in the analysis because these data may not directly reflect program use (eg, program completion), but rather rates of study participants who did not fulfill the research protocol (eg, filling out questionnaires) [15]. Corresponding authors were contacted to provide clarity and completeness of unclear or unreported information and to ensure accurate application of the PSD model for coding iCBT program features. An author for each of the included programs responded to our requests (n=10).

### Data Analysis and Synthesis Process

We used a multistep approach to data analysis that was structured according to Pawson’s techniques [27,28] and meta-ethnography [43,44]. The first step involved determining recurrent patterns or themes (demi-regularities) across documents for each iCBT program for delivery Context, PSD features and program Mechanisms, and Outcomes related to program use. The purpose was to use evidence from the literature to (1) identify PSD Mechanisms in each program most frequently associated with the program use Outcomes to refine the candidate Mechanism-Outcome dyads, and (2) incorporate delivery Context of each iCBT program into the dyads to refine the overall Context-Mechanism-Outcome configurations. Context-Mechanism-Outcome configurations that were supported by evidence from at least two different iCBT programs progressed to the next step of analysis.

The second step in the analysis involved reciprocal translation analysis, a meta-ethnographic technique that involved reviewing the Context-Mechanism-Outcome configurations across iCBT programs [44,45]. Configurations that were found to have mixed (ie, more heterogeneous support with no larger trend) or confounding evidence across programs, or could not be refined by better describing or recombining the Context, Mechanism or Outcome factors, did not progress to the next stage of analysis. What remained were configurations that provided the best support, across multiple programs, to explain the relationship of design and delivery components of iCBT with program use.

In the final step, we used lines-of-argument synthesis, a theorizing technique [44] that involved assessing how well each Context-Mechanism-Outcome configuration could explain why the same PSD Mechanism(s), operating in different iCBT program Contexts, might result in particular program use Outcomes. We took into consideration the quality and quantity of evidence supporting the configuration and held a meeting to discuss, amend, and finalize configurations with individuals from across Canada with expertise in electronic health interventions. Configuration refinement continued until we felt that it reflected a pattern that would remain consistent despite differences in small- or large-scale details across iCBT programs. At that point in time, we considered the configuration to be adequately developed.

## Results

### Included Documents

**Figure 1:**
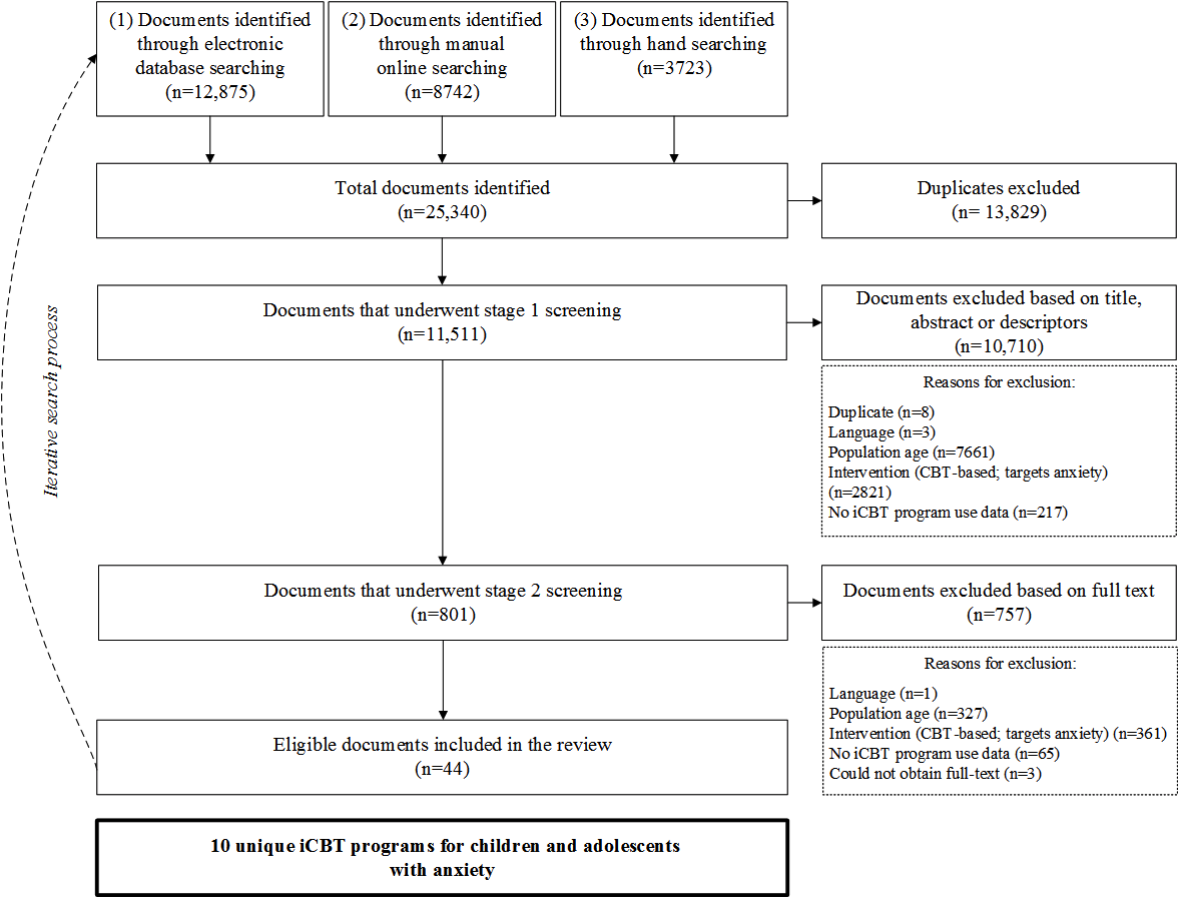
Flow diagram of the literature search and selection process. iCBT: internet-based cognitive behavioral therapy.

The literature search and selection progress are presented in Figure 1. The search strategy yielded 11,511 unique documents for stage 1 screening, after duplicates were removed. Of these, 801 documents underwent stage 2 screening. In total, 44 documents detailing 10 iCBT programs were included in the realist synthesis. Documents were published studies (n=20), theses (n=5), published or registered protocols for trials (n=12), study or program websites (n=6), and a study flyer (n=1).

### Characteristics of Internet-Based Cognitive Behavioral Therapy for Anxiety in Children and Adolescents

#### Program and Participant Characteristics

Table 1 presents an overview of iCBT program and user characteristics. The majority of child and adolescent users were white, English speakers, of middle-to-high socioeconomic status, who lived in urban centers with both biological parents. Programs designed to treat an anxiety disorder tended to be longer in duration (included more modules) than prevention-based programs. Treatment-based programs were delivered in the community (some included occasional health care clinic visits) and involved weekly Web- or email-based therapist interaction and parent-dedicated modules. Prevention-based programs were often provided in schools, with a teacher facilitating program administration. Most iCBT programs were adaptations of previously developed mental health prevention or treatment resources [46-55].

**Table 1:** Overview of the internet-based cognitive behavioral therapy user, program, and delivery characteristics.

Numbered list of programs^a^		User details	Delivery		Therapist support in program			Adjunct support
			Setting	Number of modules and duration	Web or email	Phone	In-person	
**Treatment programs**								
	1. BRAVE-ONLINE	Children and adolescents with anxiety	Clinic or community	10 weekly modules + 2 booster modules, 60 min each	✓	✓	—^b^	Parent
	2. iCBT^c^ for children and adolescents with dental anxiety	Children and adolescents with anxiety	Community plus clinic	12 weekly modules	✓	—	—	Parent, Dental professional^d^
	3. Internet-delivered CBT for children with anxiety disorders	Children with anxiety	Community	11 modules over a 10-week period^e^	✓	✓	—	Parent
	4. Internet-delivered CBT for children with specific phobia	Children with anxiety	Community	11 modules over a 6-week period, 15-45 min each	✓	✓	—	Parent
	5. SmartCAT App for children with anxiety disorders	Children with anxiety	Community	Daily app entries completed over 8 in-person modules, 3-4 min each	✓	—	✓	Parent
**Indicated prevention programs**								
	6. Internet cognitive behavioral skills-based program	Children with anxiety	Community	3 modules over a 12-week period^f^	—	✓^g^	—	Parent
	7. REACH for success app^h^	Children with anxiety	School	5 activities, 20-30 min for each activity	—	—	✓	Research assistant^i^
	8. Individually tailored iCBT for adolescents^h^	Adolescents with anxiety, or anxiety and depression	Clinic	6-9 prescribed modules over a 6-18-week period^j^	✓	✓	✓	Therapist (optional)
**Universal prevention programs**								
	9. The e-couch anxiety and worry program	Adolescents with anxiety	School	6 weekly modules, 30-40 min each	—	—	—	Teacher^k^, Mental health service provider^l^
	10. MoodGYM	Adolescents with anxiety, or anxiety and depression	School or community	5 weekly modules, 30-60 min each	—	—	—	Teacher^k^

^a^Categorized according to the Level of Prevention Model [41]: universal prevention: target participants have not been identified on the basis of individual risk (ie, no symptoms required); selective prevention: target participants have a higher risk of developing an anxiety disorder than others; indicated prevention: target participants are high risk, and who have anxiety signs or symptoms, but do not currently meet diagnostic levels; and treatment: target participants are diagnosed with an anxiety disorder.

^b^N/A: not applicable.

^c^iCBT: internet-based cognitive behavioral therapy.

^d^A dental professional (a dentist, dental hygienist, or dental assistant) provided exposure at a dental clinic.

^e^Five versions depending on diagnosis.

^f^Two blocks of modules (containing multiple sections) dedicated to mothers and 1 module block (containing multiple sections) dedicated to child + mother.

^g^Therapist completed a brief (15 min), nontherapeutic, check-in telephone call with the mother (not the child).

^h^Program was designed for indicated prevention or treatment (early intervention).

^i^Research assistant or graduate student was present to facilitate aspects of the study such as assessment and troubleshoot technical issues.

^j^Out of a possible 17 modules, based on symptoms.

^k^Teacher facilitated program administration and was available for general guidance or if questions arose but did not provide an active therapeutic role.

^l^Mental health service provider was present in 1 study of the program to facilitate program administration or address student questions [56].

**Figure 2:**
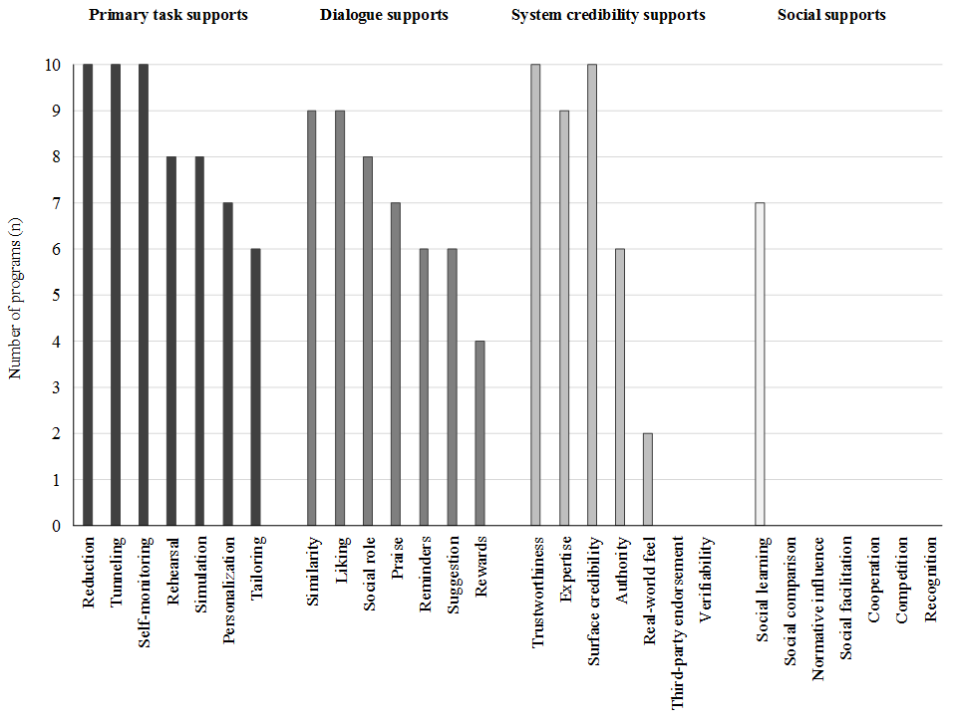
Overview of the persuasive systems design features across the 10 internet-based cognitive behavioral therapy programs included in the synthesis.

#### Cognitive Behavioral Therapy Components and Persuasive Systems Design Features

All programs described themselves as CBT-based and contained at least 3 of the 5 AACAP-recommended CBT components (most commonly psychoeducation, somatic management skills, and cognitive restructuring). The workflow of the programs presented the more foundational and simpler treatment components (eg, psychoeducation and symptom identification) before more challenging skills (eg, desensitization or exposure). Many programs also integrated interpersonal therapy skills [57], such as assertiveness training and problem solving, to reduce environmental stressors and enhance social support.

The frequency of the PSD features used in the iCBT programs is shown in Figure 2. All programs incorporated Reduction and Tunneling (Primary task supports) to regulate the pace and sequence by which users moved through the program. Self-monitoring, a Primary task support, was also used in all programs to track users’ progress or mood over time. A total of 8 programs used Rehearsal by providing recommended or required Web-based homework or practice activities. Moreover, 7 programs provided a Personalized review (eg, progress report and feedback) of homework or module content before the next module was accessible to users. Most programs used Tailoring (n=6) or Personalization (n=7) (Primary task supports) to adapt the content to users’ characteristics such as their primary anxiety concern, age, or name. Incorporating a Social role, such as a Web-based or virtual therapist or cartoon guide, was one of the most commonly used Dialogue supports (n=8). In addition, 9 of the 10 programs had program content, technology, and interaction features that were relatable and appealing to target users (Similarity and Liking features). As all programs were part of a research study, Trustworthiness and Surface credibility (System credibility supports) were considered inherent to their design (ie, programs were ad-free, not marketed for commercial use, and accessed through a secure platform), although few documents explicitly reported this. Authority (System credibility support) was incorporated when Web- or email-based therapist support was provided. Social support features were seldom used among programs.

### Level of Contribution and Methodological Quality

Details of the quality appraisal are provided in Multimedia Appendix 4 [58-75]. Documents tended to provide the greatest level of contribution to understanding program Context and Outcomes with relatively few details available for understanding program Mechanisms. A total of 7 iCBT programs had at least one document that scored *high* for level of contribution for program Context, Mechanisms, and Outcomes. We evaluated 29 research studies (found in 25 documents) for methodological rigor. A total of 19 studies met all 4 MMAT criteria (100%), 7 met 3 criteria (75%), and 3 met 2 criteria (50%). Lower ratings reflected an unclear description of: processes for recruitment, selection, randomization, or allocation; how group differences (if any) were controlled for; the percentage of outcome data obtained; or withdrawal or dropout rates were not within acceptable limits.

### Summary of Key Internet-Based Cognitive Behavioral Therapy Contexts, Mechanisms, and Outcomes

Tables 2 and 3 present an overview of the delivery Context and PSD Mechanisms that were most frequently or consistently associated with moderate-to-high program use Outcomes across iCBT programs. Contexts most indicative of program use were the adjunct support person and the communication approach (eg, Web [the program platform or internal messaging system], email, or in-person) provided to the user—both of which typically varied based on the level of prevention the program was designed for. The type of adjunct support also depended on the age of program users. Children generally received the most extensive adjunct support (ie, therapist and parent), and program use was often greater among this age group than among adolescents. PSD features identified as having a high value to encouraging program use were Tailoring and Personalization (Primary task supports); Social role, Reminders, and Rewards (Dialogue supports); and Authority, Expertise, and Trustworthiness (System credibility supports). Program use Outcomes most commonly reported either the total proportion or average proportion of program modules, homework, or activities completed by users. More than half of the Outcomes indicated *high* or *moderate* program use.

When finalizing our proposed configurations, we considered both what program Contexts and Mechanisms were combined (using Tables 2 and 3) and in what way and for what purpose they may have been combined (using excerpts from individual documents; see Table 4) to explain how moderate-to-high program use Outcomes were generated.

### Proposed Internet-Based Cognitive Behavioral Therapy Contexts and Mechanisms for Moderate-to-High Program Use

Key Contexts and PSD Mechanisms that may have led to moderate-to-high iCBT program use Outcomes are described in Table 4 alongside examples from contributing programs.

#### Context-Mechanism-Outcome Configuration 1: Tailoring and Personalization

Evidence from 8 iCBT programs suggested that indicated prevention and treatment programs that provided adjunct support along with Tailoring and Personalization resulted in greater program use. Studies supporting this configuration had a mean MMAT score of 90.2%.

Tailoring and Personalization were used to provide users with more information to increase the individualized feel of the program and portray that the program knew and could meet the user’s needs. Programs most commonly tailored content (ie, therapeutic elements and examples) based on the user’s age or mental health condition (eg, specific phobic and social anxiety). User information was often collected by the adjunct support person during study enrollment (ie, part of eligibility screening). Personalization was a feature that could be initiated through program automation (ie, user’s name appeared on the home screen; his or her pronouns) or via program communications (ie, individualized weekly emails and secure messages). The adjunct support person acted as an extension of the Tailored or Personalized program experience through their contact with users within (eg, by providing personalized feedback on Web-based homework [50,76,83]) or outside of the iCBT program (eg, by tailoring in-person session objectives [55]). Programs with a combination of Tailoring and Personalization reported some of the highest program use Outcomes.

#### Context-Mechanism-Outcome Configuration 2: Reminders

Evidence from 6 iCBT programs suggested that indicated prevention and treatment programs that provided adjunct therapist support along with Reminders also resulted in greater program use. Studies supporting this configuration had a mean MMAT score of 92.1%.

The programs contributing to this Context-Mechanism-Outcome configuration involved multiple modules or activities (the number of modules ranged from 6-11 or app use ranged from daily-weekly activities); therefore, users were required to log into the program over numerous instances. Reminders were used to encourage the user to take program action, either by promoting skills practice [46] or by “prompt[ing] participants who [were] late in completing a module” [78] to access the newly available content. Programs provided generic and automatized Reminders through email or the Web-based platform. Reminders were also embedded in the regular, electronic, personalized communications (eg, feedback and progress check-ins) sent by the adjunct therapist to the child or adolescent. If users remained absent from the program beyond the recommended treatment schedule (eg, longer than 1 week), the adjunct therapist provided additional in-person or telephone follow-up, encouraging users to access the next available module.

#### Context-Mechanism-Outcome Configuration 3: Rewards

Evidence from 4 iCBT programs suggested that programs and mobile apps that provided adjunct support along with Rewards resulted in greater program use. Studies supporting this configuration had a mean MMAT score of 85.0%.

**Table 2:** An overview of the delivery Context and persuasive systems design features that may explain program use Outcomes across internet-based cognitive behavioral therapy treatment programs.

Program and document		Context: Target users and adjunct support	Mechanism: PSD^a^ features	Outcome: Posttreatment findings (program use summary^b^)
**Program 1: BRAVE-ONLINE for children and adolescents with anxiety disorders**				
	[49]	Child users; Therapist support: in-person, Web, email, phone; Parent support: in-person, module	Primary task support: Tailoring and Personalization; Dialogue support: Social role and Reminders; System credibility support: Authority, Expertise, and Trustworthiness	91% of homework completed (high use)
	[76]	Child users; Therapist support: Web, email, phone; Parent support: modules	Same as above	95% of module activities completed (high use)
	[77,78]	Same as above	Same as above	Average of 7.5/10 modules completed (high use); 33.3% of users completed all 10 modules (low use)
	[79]	Same as above	Same as above	Average of 4.88/10 modules completed (low use)
	[76]	Adolescent users; Therapist support: Web, email, phone; Parent support: modules	Same as above	85% of module activities completed (high use)
	[80]	Same as above	Same as above	Average of 7.5/10 modules completed (high use); 39% of users completed all 10 modules (low use)
	[80]	Child and adolescent users; Therapist support: Web, email, phone; Parent support: modules	Same as above	Average of 7.9/10 modules completed (high use); 42.6% of users completed all 10 modules (low use); 73.5% of module tasks completed (moderate use); Treatment expectancy predicted compliance (N/A^c^)
	[81]	Same as above	Same as above	Average of 85% module tasks completed (high use); Average of 8.9/10 modules completed (high use)
	[82]^d^	Same as above	Same as above	Average of 6.7 /10 modules completed (moderate use); 19% of users completed all 10 modules (very low use)
	[23]^e^	Same as above	Same as above	Average of 4.8/10 modules completed by children (low use); Average of 4.0/10 modules completed by adolescents (low use)
**Program 2: iCBT^f^ for children and adolescents with dental anxiety**				
	[48]	Child and adolescent users; Therapist sup- port: Web; Parent support: in-person; Dental professional support: in-person	Primary task support: Personalization; Dialogue support: Social role; System credibility support: Authority, Expertise, and Trustworthiness	Average of 9.2/12 modules completed (high use)
**Program 3: Internet-delivered CBT for children with anxiety disorders**				
	[54,83]	Child users; Therapist support: Web, email, phone; Parent support: modules	Primary task support: Tailoring and Personalization; Dialogue support: Social role and Reminders; System credibility support: Authority, Expertise, and Trustworthiness	Average of 9.7/11 modules completed (high use)
	[54,84]	Same as above	Same as above	83% of users completed ≥9 of 11 modules (high use)
	[85]	Same as above	Same as above	Average of 6.0/12 modules completed^g^ (moderate use); 53% of users reached at least module 4 (first exposure exercise; moderate use)
**Program 4: Internet-delivered CBT for children with specific phobia**				
	[50]	Child users; Therapist support: Web, email, phone; Parent support: modules	Primary task support: Personalization; Dialogue support: Social role and Reminders; System credibility support: Authority, Expertise, and Trustworthiness	80% of users completed ≥9 of 11 modules (high use)
**Program 5: SmartCAT App for children with anxiety disorders**				
	[46]	Child users; Therapist support: in-person, mobile app; Parent support: in-person	Primary task support: Tailoring and Personalization; Dialogue support: Social role, Reminders, and Rewards; System credibility support: Authority, Expertise, and Trustworthiness	Average of 82.8% of practice entries completed (high use)

^a^PSD: persuasive systems design.

^b^Program use summary was calculated by dividing the reported value by 100 or converting it to a percentage. High use (≥75%), moderate use (50-74%), low use (25-49%), or very low use (≤24%).

^c^Not applicable

^d^All participants were diagnosed with a high functioning autism spectrum disorder and anxiety disorder.

^e^This study compared participants who were randomized to 1 of 2 iCBT conditions: iCBT-generic (iCBT relevant to multiple types of anxiety; ie, social, separation, and generalized anxiety) or iCBT-social anxiety (iCBT specific to social anxiety).

^f^iCBT: internet-based cognitive behavioral therapy.

^g^Data available for 15 out of 17 participants.

A total of 3 iCBT programs regularly incorporated Rewards into modules to encourage ongoing program use and promote the completion of essential treatment exercises [46,47,51]. The iCBT program for dental anxiety opted for a final Reward and presented users with a virtual diploma at the end of their treatment [45]. Rewards were also used as a proxy to track program progress, including completion of exposure activities [46,47,51]. Unlike computer-based programs, the open and flexible design approach to mobile apps gave users the option to select what treatment content and tasks they wanted to access and when. Progressive reward incentives were used to persuade users to complete more of the app’s content and critical components. In the REACH for success app, a cartoon character provided regular guidance and feedback to users and entertained them with animations following task completion (Reward) [47]. In the SmartCAT app, a point system tied to external prizes (Reward) was a feature managed by the adjunct therapist [46]. In-person sessions with a therapist or parent also provided positive reinforcement of program use (ie, Praise, Rewards); sessions were also used to instruct users on how to incorporate Rewards into their anxiety management activities outside of the program [48,51].

#### Context-Mechanism-Outcome Configuration 4: Therapist, Social Role, Authority, Expertise, and Trustworthiness

Evidence from 6 iCBT programs suggested that indicated prevention and treatment programs with adjunct Web- or email-based therapist support that also provided a Social role component, and conveyed Authority, Expertise, Trustworthiness, resulted in greater program use. Studies supporting this configuration had a mean MMAT score of 91.3%.

The Social role component of iCBT programs was often fulfilled by a therapist or coach (who received specialized training with the program but may not have been a licensed psychologist). Therapists engaged in regular, Web- or email-based communication with the user and served 2 roles by (1) facilitating program delivery by providing technical support and answering users’ questions and (2) promoting program completion by providing reminders and encouragement, reinforcing program concepts, and ensuring accurate comprehension and application of CBT skills. Together, the Social role feature and therapist emails complemented (had overlap with) other PSD features such as Reminders, Praise, and Suggestion. Therapists had secure access to users’ written responses or logged data so that they could send specific communications to users, demonstrating therapists’ credibility and competence with both the therapeutic process and individual responsiveness (Authority, Expertise, and Trustworthiness). Moreover, 3 studies measured child-reported therapeutic alliance with their iCBT program therapist and found it to be strong and program use to be high [76,81]. One of these studies correlated therapeutic alliance with program use and found a significant, positive relationship [81].

#### Context-Mechanism-Outcome Configuration 5: Therapist + Parent, Social Role, Authority, Expertise, and Trustworthiness

Evidence from 5 treatment programs suggested that iCBT programs with adjunct therapist and parent support that also included a Social role component, and conveyed Authority, Expertise, and Trustworthiness, had greater program use. Studies supporting this configuration had a mean MMAT score of 90.8%.

**Table 3:** An overview of the delivery Context and persuasive systems design features that may explain program use Outcomes across internet-based cognitive behavioral therapy indicated prevention and universal prevention programs.

Program and document			Context: Target users and adjunct support	Mechanism: PSD^a^ features	Outcome: Posttreatment findings (program use summary^b^)
**Indicated prevention programs**					
	**Program 6: Internet cognitive-behavioral skills-based program**				
		[51]	Child users; Therapist support: phone; Parent support: modules	Primary task support: Tailoring; Dialogue support: Rewards; System credibility support: Trustworthiness	Average of 82.6% modules completed; Users who immediately accessed the program completed more sections (average=17.35) than those who had delayed access (average=8.0); Immediate access users spent more time in the program (average=183.3 min) than those who had delayed access (average=77.6 min); Use time was positively correlated with number of sections completed (high use)
	**Program 7: REACH for success app**				
		[47]	Child users; Therapist support: in-person	Primary task support: Tailoring and Personalization; Dialogue support: Social role, Reminders, and Rewards	93.2% of users completed relaxation practice (high use); 91.7% of users completed hypothetical cognitive self-control practice (high use); 15.2% of users completed applied (very low use) cognitive self-control practice (very low use); 45.5% of users completed self-monitoring (low use); The proportion of users who attempted an activity was higher than those who completed an activity (N/A^c^)
		[47]	Same as above	Same as above	Users completed more activities before an evaluation module (N/A); App use was highest in week 1 and decreased over 6 weeks (N/A); 100% of users completed re-laxation practice (high use); 100% of users completed hypothetical cognitive self-control practice (high use); 60.0% of users completed self-monitoring (moderate use); 0% of users completed exposure practice (very low use)
	**Program 8: Individually tailored iCBT^d^** **for adolescents**				
		[55, 86]	Adolescent users; Therapist support: in-person, email, phone	Primary task support: Tailoring and Personalization; Dialogue support: Social role and Reminders; System credibility support: Authority, Expertise, and Trustworthiness	Average of 6.5/9 modules completed (moderate use)
**Universal prevention programs**					
	**Program 9: The e-couch anxiety and worry program**				
		[87]	Adolescent users; Teacher support: in-person	Dialogue support: Social role	45% of users completed all modules (low use)
		[24]	Same as above	Same as above	50% of users completed all modules (moderate use)
		[24]	Adolescent users; Teacher support: in-person; Mental health provider support: in-person	Same as above	36% of users completed all modules (low use)
	**Program 10: MoodGYM**				
		[53]	Adolescent users; Teacher support: in-person	Dialogue support: Social role	Average of 3.2/5 modules completed (moderate use)
		[25]	Same as above	Same as above	Average of 9.4/28 exercises completed (low use); >25% of users completed all modules (low use)
		[25]	Adolescent users	Same as above	Average of 3.1/28 activities completed (very low use)
		[26]	Adolescent users; Teacher support: in-person	Same as above	<1% of users completed all activities (very low use)

^a^PSD: persuasive systems design.

^b^Program use summary was calculated by dividing the reported value by 100 or converting it to a percentage. High use (≥75%), moderate use (50-74%), low use (25-49%), or very low use (≤24%).

^c^Not applicable.

^d^iCBT: internet-based cognitive behavioral therapy.

**Table 4:** Configuration summaries of the key Contexts and persuasive systems design Mechanisms that may have led to moderate-to-high program use Outcomes.

Context	Mechanism				Program #
	PSD^a^ feature(s) and proposed purpose		Example		
Indicated prevention and treatment programs with adjunct support	Configuration 1: Tailoring +/or Personalization to increase relevance of program content	Through email the therapist provided “written feedback on worksheets” and was available to “answer questions and clarify treatment content, increase motivation and to help solve problems” [83]. A participant’s name was populated in modules and feedback messages [76].			1, 2, 3, 4, 5, 6, 7, and 8
	Configuration 2: Reminders to increase awareness of program availability and progress	“Participants receive automated, computer-generated, standardized, weekly e-mails both before each module (as a reminder to complete their modules) and after each module (to congratulate them on finishing their module)” [78]. “Each skills coach entry ends with a customized motivational message from the therapist (entered weekly via the [app] portal) that includes encouragement as well as a reminder to complete any assigned home-based exposure or skills practice” [46].			1, 3, 4, 5, 7, and 8
	Configuration 3: Rewards to recognize and encourage achievement	Following task completion, the user received a reward in the form of Bob’s abilities or tricks, with more complicated tricks being unlocked as users completed more of the treatment protocol [47]. Program progress was presented and tracked with a virtual sticker chart. A cartoon figure would jump to the next rung of the ladder indicating successful completion of an exposure hierarchy activity [51].			2, 5, 6, and 7
Indicated prevention and treatment programs with adjunct therapist support	Configuration 4: Social role to increase program interaction; Authority+Expertise+Trustworthiness to improve perceived value of information or support	Participants received “comments and feedback from their therapist on all exercises, and the technical platform also allowed participants to comment on worksheets” [50]). The therapist portal and secure messaging features in the app allowed the participants and therapist to securely exchange information such as messages, documents, or audio or video files related to treatment [46].			1, 2, 3, 4, 5, and 8
Treatment programs with adjunct therapist support plus parent support	Configuration 5: Social role to increase program interaction; Authority+Expertise+Trustworthiness to improve perceived sense of reliance and cooperation toward program progress	Parents were provided with their own modules during treatment. “In this way, the parent was empowered to help their child acquire and use the skills presented in the program, and to handle situations in which their child became anxious” [77]. Check-in telephone calls from the therapist consisted of 4 elements: (1) progress updates, (2) symptom assessments, (3) encouragement to use the program, and (4) troubleshooting [51].			1, 2, 3, 4, and 5

^a^PSD: persuasive systems design.

Treatment-based iCBT programs were designed to be child-parent combined or parent-supported child-based interventions. Combined interventions required parents to complete parent-specific modules (eg, psychoeducation, relaxation training, problem solving, and modeling adaptive behaviors) either before or alongside their child as they completed their own child-directed modules. In parent-supported interventions, parents may have also been responsible for explaining program instructions and assisting their children with their modules [50,54,76], coaching or supporting their child with in vivo exposure or practice activities [46,48,50], and overseeing their child’s treatment schedule [54]. The support the adjunct therapist provided to children was also extended to parents. Parents had the opportunity to ask clarifying questions, receive expert advice, and troubleshoot difficulties with their child’s iCBT progress with the program therapist. Studies found that both parent and child ratings of therapeutic alliance and program use were high [76,81]. One study correlated therapeutic alliance and program use and found significant, positive relationships for both parents and children [81]. Anderson et al [81] hypothesized that therapist emails may have contributed to fostering a strong therapeutic alliance.

## Discussion

### Principal Findings

The role of technological, persuasive components in iCBT programs is an understudied aspect of program design and evaluation. The extent of iCBT program use may be a fundamental indication of its *persuasiveness* and its potential to assist the user with their goals of the program [15,88-91]. This realist synthesis identified 5 possible relationships as to how the use of specific PSD features (technological Mechanisms), supported by some key user and delivery features (Context), may generate moderate-to-high program use (Outcomes) in iCBT for children and adolescents with anxiety.

The 5 Context-Mechanism-Outcome configurations provide support for several persuasive strategies to improve iCBT program use: Tailoring and Personalization as Primary task supports; Rewards, Reminders and Social role features in programs serving as Dialogue supports; and Trustworthiness, Expertise, and Authority features serving as System credibility supports for a program. Traditionally, PSD features that stimulate human-computer communication, such as Dialogue supports, have been the most widely used and studied features for improving program use [6,15,89,92,93]. However, this synthesis demonstrated that having credibility (System credibility supports) and supporting users in completing their target behavior (Primary task supports) may also promote moderate-to-high program use. We hypothesize that using multiple PSD features, both within and across the different support categories, may produce additive or synergistic effects on program use; however, there was insufficient evidence available for our analysis to explain the impact of PSD feature combinations that involved more than a few features (and proposed functions) at a time. This is because the authors of the original studies included in our review typically discussed or formally tested the relationship of only 1 or 2 PSD features and program use at a time. Therefore, our configurations present the fewest possible PSD features that could be linked to higher program use (ie, we uncoupled features as much as possible—an approach that may make testing of their effects more efficient in future studies).

Moreover, we suspect that not all PSD features may have equal influence on program use. Depending on the program, some PSD features may be *necessary* for program use (part of the basic requirements or foundational design framework of iCBT), whereas others may be *complementary* to program use (have an impact by enhancing the design framework of iCBT); although both types of features together may influence program use. In this realist synthesis, all 10 iCBT programs described a purposeful design that incorporated Reduction and Tunneling (Primary task supports) and Similarity and Liking (Dialogue supports) to create a logical, incremental, relevant, and aesthetically pleasing experience for users—these may be the *necessary* PSD features for iCBT for children and adolescents with anxiety. The PSD features described in our configurations are hypothesized to be *complementary—*building on the persuasiveness of necessary PSD features to further improve or optimize iCBT program use.

A meta-analysis of PSD features used in internet-based interventions for mental health demonstrated that determining the amount and type of persuasive principles to include may be a delicate balance, as some principles seem to work together, whereas when other principles co-occur, they may have an unapparent or diminishing effect [90]. As was found for this synthesis, it is not necessarily the number of PSD features used in iCBT, but it is their proposed purpose or implementation that is particularly critical for optimizing program use outcomes [90]. For example, when comparing 2 indicated prevention programs, we observed that the internet cognitive behavioral skills-based program [51] had fewer PSD features than the individually tailored iCBT program [55], although the former reported greater program use. At this time, our understanding of how to best bring together PSD features, such as Personalized Reminders [93], in the design and delivery of iCBT for child and adolescent anxiety is limited. Therefore, further research on the theory, function, quality, and effectiveness of individual PSD features is needed to deliberately use and combine them for idealized treatment outcomes. Moreover, involving target users in the (co)design and testing of treatments is recommended to improve the acceptability, feasibility, and effectiveness of iCBT with children and adolescents [19,94,95]. These participatory research efforts may provide important guidance on the usefulness and functionality of select (PSD) features of iCBT programs in the *real world* and from the user’s perspective [96]; therefore, facilitating greater program use [97].

In this synthesis, 3 important potential relationships were identified: (1) adjunct support seemed to improve program use even when input or support was minimal (eg, in-person, classroom-based program administration with no treatment advice given) or when it was provided by a nonexpert (eg, teacher) [24,25]; (2) users of treatment programs demonstrated higher program use than users of universal prevention programs; and (3) a trend for increased program use among programs for children (more so than for adolescents) was identified. Within these relationships, multiple contextual aspects or user characteristics may have also had an additive or synergistic effect on program use. For example, the level of expertise the adjunct support person had (eg, teacher vs therapist) and the degree of their guidance or therapeutic involvement (eg, in-person program administration vs personalized feedback emails) increased from prevention-based to treatment programs. In the literature, little is known about how much, when, and what type of support is necessary for enhancing program use and efficacy [98,99]. Although some evidence suggests that layperson support is as effective as clinician support [100], this synthesis suggests that the person providing support as well as the intensity of their support activities (frequency; inclusion of therapeutic elements) may have a noteworthy effect on program use. The nature of the role adjunct support plays in iCBT program use is also unclear. It has been suggested that adjunct support may leverage the advantages of therapeutic alliance [101,102], which might include principles of persuasion (eg, users feel the need to respond to social cues [18]), it may establish process expectations and social accountability [103], or it may develop a sense of legitimacy or credibility of the program [103] (see Santarossa et al’s study [104] for further suggestions). Programs that had both therapist and parent involvement may have (1) reinforced child’s understanding of and confidence in treatment content and (2) increased the child’s interaction with the program by creating a perceived sense of cooperation (shared goals) and accountability toward treatment progress. In this realist synthesis, adjunct support may have been used to complement or replace the use of some PSD features in iCBT, particularly Dialogue supports. For example, in-person therapist sessions or telephone calls provided opportunities for Reminders, Personalized feedback, or Praise to be conveyed to users [49,50,54,55]. Consideration of how and when to provide adjunct support is critical when preparing for the implementation and integration of iCBT within routine practice, such as allotting for therapist time, making changes in clinical workflow, and when conducting economic analyses.

### Future Directions

This realist synthesis not only provides support for incorporating some of the well-studied and highly used PSD features into iCBT (ie, Reminders [93,105,106]) but also draws attention to underutilized features that can be incorporated in the designs of new treatments. For example, Rewards were only occasionally used by iCBT programs included in this study (4 out of 10 programs) but are more commonly used techniques for increasing program use in internet-based interventions targeting physical activity or dietary behaviors [107]. Recent efforts into improving the gamification of technology-based CBT for pediatric mental health (see SPARX [108]), where incorporating game-design elements such as Praise and Rewards are regularly used to enhance program use and engagement [109], demonstrate the potential benefits that the use of these features may have.

Although this synthesis and other recent reviews have been helpful for identifying PSD features of interest for improving program use of internet-based interventions [15,89,90], the next step is to formally isolate and evaluate the effectiveness of these PSD Mechanisms in producing optimal program use. This synthesis suggests 8 features that may be a priority for further examination. Modeling, factorial designs or the multiphase optimization strategy [110-112] (see also Baker et al’s study [113] for other suggestions) can be used to evaluate the best set (individual or combination) of program features to use under different conditions (eg, delivery setting and start or end of treatment). Studies with multiple, active treatment arms would also allow the examination of the comparative effects of select program features [114] or in different delivery contexts (ie, varying the type of adjunct support). From this synthesis, only 3 studies of 3 different programs conducted these comparisons. These studies provided important insights into the impact of delivery medium [49], type of adjunct support [24], and delivery location [25] on iCBT program use. Qualitative studies or self-report data would also provide meaningful information on the factors affecting program use from child and adolescent or health care provider standpoints. Another important line of inquiry relates to defining and measuring program use to ensure its validity and reliability for future studies. Designing studies that incorporate in vivo, objective measurements or automatic data capture of program use [115,116] could improve our awareness of program use predictors beyond user demographics (ie, age), for example, to actual usage behaviors (ie, number of Web pages viewed). This method would allow for iCBT program use to not only be measured at end of the intervention but also throughout the program access period to assess usage patterns over time [15], when certain design or delivery features may be more or less *activated* or present.

### Strengths and Challenges of Realist Synthesis

This is the first study to examine PSD features as they relate to program use in iCBT for children and adolescents with anxiety. A strength of this synthesis is the inclusion of diverse and high-quality evidence (ie, MMAT scores>75% [38]) from both the published and gray literature. Approaching our research questions using a single theoretical framework (ie, the PSD model) allowed for systematic and incremental accumulation of knowledge about how iCBT may work from a trackable, technological perspective.

The lack of operationalization of how PSD features and aspects of Context and program use Outcomes were defined, described, and measured by authors affected our data extraction and coding strategies. As adherence to recent recommendations [19] and reporting guidelines, such as Consolidated Standards of Reporting Trials-electronic health (CONSORT-EHEALTH) [117], become mandatory for publication, the opportunity to identify the active ingredients of iCBT will improve. Clarifying PSD features with original authors was an attempt to mitigate the potential bias that lies in coding technological program features and interpreting the papers using the PSD model [92]. However, few details about the time or the quality of communication by the adjunct support person(s) were available, limiting our understanding of the important role this contextual feature played in program use. For reasons of inclusivity, we described the heterogeneous outcomes using the umbrella term *program use*. Adoption of a recent standardized definition and calculation of *adherence* [118] can clarify what is meant by specific program use terms and allow for comparisons of outcomes across programs. iCBT programs’ widespread implementation and ability to meet the health goals of users will involve an understanding of the expectations and actuality of program use in the real world (ie, using true effectiveness studies or formative program evaluation), and setting benchmarks for an *effective dose* in different delivery settings. Finally, like others [119-121], we recognized the outcomes of persuasive systems depended on multiple factors, many of which were not examined in this synthesis. However, it was rare to have information on treatment or technology preferences of users (eg, early completers [22,122]), their psychological characteristics or cognitions (eg, motivation, personality, expectations, and treatment perceptions [123]), or personal circumstances (eg, program access [124])—factors that are also considered critical to program use and could be used to construct and validate more intricate Context-Mechanism-Outcome configurations.

### Conclusions

The Context-Mechanism-Outcome configurations identified by this realist synthesis provide an initial understanding of how, why, and for whom iCBT programs for children and adolescents with anxiety work from a persuasive systems’ perspective. Appreciating that the effectiveness of iCBT programs may hinge on whether and to what extent programs are used, this study is an important step toward successfully implementing and integrating iCBT into routine clinical care. Recognizing that multiple PSD features are incorporated in iCBT program designs and that individual features may affect each other differently, further knowledge and testing of the purpose and function of these features will help determine the number and combination to use in certain delivery contexts (eg, adjunct support included; level of prevention a program is designed for). As PSD features are modifiable, iCBT program designers and developers can look to create more persuasive programs that promote greater use and improved treatment outcomes.

## Appendix

Multimedia Appendix 1 Candidate Context-Mechanism-Outcome configurations.

Multimedia Appendix 2 Document electronic search strategy.

Multimedia Appendix 3 The persuasive systems design (PSD) model.

Multimedia Appendix 4 The level of contribution and methodological quality of documents for the included internet-based cognitive behavioral therapy programs.

## Abbreviations

AACAP: American Academy of Child and Adolescent Psychiatry
CBT: cognitive behavioral therapy
CIHR: Canadian Institutes of Health Research
CONSORT-EHEALTH: Consolidated Standards of Reporting Trials-electronic health
iCBT: internet-based cognitive behavioral therapy
MMAT: mixed method appraisal tool
PSD: persuasive system design
