# Supplementary material for: Design and Delivery Features That May Improve the Use of Internet-Based Cognitive Behavioral Therapy for Children and Adolescents With Anxiety: A Realist Literature Synthesis With a Persuasive Systems Design Perspective
Source: J Med Internet Res. 2019 Feb 5;21(2):e11128. doi: 10.2196/11128 (PMC6379818; doi:10.2196/11128)
Supplement: Multimedia Appendix 1 [file jmir_v21i2e11128_app1.pdf]

Multimedia Appendix 1. Candidate Context-Mechanism-Outcome configurations.

**Context**	**PSD Mechanism**	**Program use Outcome**	**Examples of literature used to develop the configuration**
iCBT programs with adjunct therapist, parent and/or professional support	Primary task support features that simplify tasks or streamline the program content for users; ie, Reduction, Tunneling	High	[18, 30]
iCBT programs with adjunct therapist, parent and/or professional support	Primary task support features that adapt program content to the users' needs or preferences; ie, Tailoring, Personalization	High	[31, 32]
iCBT programs with adjunct therapist, parent and/or professional support	Dialogue support features that provide program progress, feedback or cues to users; ie, Reminders, Suggestions, Social role	High	[33, 105]
iCBT programs with adjunct therapist, parent and/or professional support	Dialogue support features that provide incentives or positive reinforcement to users; ie, Praise, Rewards	High	[22, 34]
iCBT programs with adjunct therapist, parent and/or professional support	Social support features that motivate users by leveraging social influence; ie, Social learning	High	[15, 103]

PSD: persuasive systems design
