# Supplementary material for: Design and Delivery Features That May Improve the Use of Internet-Based Cognitive Behavioral Therapy for Children and Adolescents With Anxiety: A Realist Literature Synthesis With a Persuasive Systems Design Perspective
Source: J Med Internet Res. 2019 Feb 5;21(2):e11128. doi: 10.2196/11128 (PMC6379818; doi:10.2196/11128)
Supplement: Multimedia Appendix 2 [file jmir_v21i2e11128_app2.pdf]

Multimedia Appendix 2. Document electronic search strategy.

1. Cellular Phone/
2. Computers/
3. Electronic Mail/
4. Internet/
5. Text Messaging/
6. Therapy, Computer-Assisted/
7. (android* or apps or cell* phone* or cellphone* or computer* or digital* or e health* or ehealth* or e mail* or email* or electronic mail* or internet* or iPad* or iPhone* or iPod* or m health* or mhealth* or mobile* or online* or personal digital assistant* or short messag* or smart phone* or smartphone* or technolog* or text messag* or virtual* or web*).tw.
8. or/1-7 [Combined MeSH and keywords for internet based technologies]
9. Behavior Therapy/
10. exp Cognitive Therapy/
11. Disease Management/
12. exp Directive Counseling/
13. Persuasive Communication/
14. Problem Solving/
15. Psychotherapy/
16. Self Care/
17. acceptance adj commitment therap*.tw.
18. (behavio* adj (activation or condition* or modif* or therap*)).tw.
19. CBT.tw.
20. cognitive therap*.tw.
21. directive counsel*.tw.
22. interpersonal therap*.tw.
23. mental health program*.tw.
24. mindfulness.tw.
25. motivational interview*.tw.
26. problem solving*.tw.
27. psychotherap*.tw.
28. (selfcar* or self car* or selfhelp* or self help* or selfmanage* or self manage* or selfmonitor* or self monitor*).tw.
29. telemental health*.tw.
30. or/9-29 [Combined MeSH and keywords for CBT]
31. Anxiety/
32. Anxiety Disorders/
33. Depression/
34. Depressive Disorder/
35. Mental Disorders/
36. Mood Disorders/
37. (anxi* or behavio?r* disorder* or behavio?r* problem* or depress* or mental disorder* or mental health* or psychiatric illness* or psychiatric disease* or psychiatric disorder*).tw.
38. or/31-37 [Combined MeSH and keywords for anxiety]
39. and/8,30,38 [Combined results for internet based technologies, CBT and anxiety searches]
