# Supplementary material for: Design and Delivery Features That May Improve the Use of Internet-Based Cognitive Behavioral Therapy for Children and Adolescents With Anxiety: A Realist Literature Synthesis With a Persuasive Systems Design Perspective
Source: J Med Internet Res. 2019 Feb 5;21(2):e11128. doi: 10.2196/11128 (PMC6379818; doi:10.2196/11128)
Supplement: Multimedia Appendix 3 [file jmir_v21i2e11128_app3.pdf]

Multimedia Appendix 3. The persuasive systems design (PSD) model.

PSD feature	Description	iCBT program example
**Primary task supports**		
Reduction	Reduces complex behaviour into simple tasks	Development and use of graded exposure activities over the course of the program
Tunneling	Guides a user through a process or experience	Allows users to access program sessions in a specific, predetermined sequence
Tailoring	Tailors the experience to the potential needs, interests, personality or use context	Information entered in at program start (e.g., primary anxiety concern) determines program content stream
Personalization	Personalizes content	Displays user's name in a welcome message at the start of every program session
Self-monitoring	Keeps track of the user's performance or status towards goal achievement	Graphs user's self-reported symptom changes over the course of the program
Simulation	Provides simulations to enable the user to observe link between cause and effect	Interactive diagram of the relationship between anxiety symptoms and anxious behaviour
Rehearsal	Provides a way for the user to rehearse a skill or task	Post-session online homework activities
**Dialogue supports**		
Praise	Offers praise as a form of feedback	Congratulatory pop-up message appears after session completion
Rewards	Rewards target behaviour	User collects points that can be applied to 'reward bank' items following program achievements
Reminders	Reminds the user of their target behaviour	Weekly email prompts user to login and access next available session
Suggestion	Offers fitting suggestions	Information for local mental health supports is provided if user reports a deterioration in mood
Similarity	Reminds the user of themselves in some meaningful way	Age appropriate phrases, metaphors and imagery are used
Liking	Is visually attractive for the user	Multimedia features are incorporated into the program
Social role	Adopts a social role	An internal messaging system for user-therapist communication

**System credibility supports**		
Trustworthiness	Provides information that is truthful, fair and unbiased	Program provides links to reliable resources and references of evidence-based treatment content
Expertise	Provides information showing knowledge, experience, and competence	Online therapist establishes rapport with user and provides specialized feedback
Surface credibility	Has a competent look and feel	Program is ad-free and appealing with a well-organized layout
Real-world feel	Provides information of the actual people behind its content and services	A Contact page with a description of research and/or clinical team
Authority	Refers to people in the role of authority	Introduces therapist with education and credentials
3rd party endorsement	Provides endorsements from other sources	Includes logos from funders and/or other affiliations
Verifiability	Provides means to verify the accuracy of the program via outside sources	Provides scientific references from which program content is based on
**Social supports**		
Social learning	Can use the system to observe others performing tasks or behaviour	Video demonstration of a teen practicing relaxation strategies
Social comparison	Can use the system to compare their performance to performance of others	Provides results of standardized questionnaires using normed data
Normative influence	Leverages normative influence or peer pressure	Testimonials from teens who report improvements after using the program
Social facilitation	User discerns via the system that others are performing the behaviour along with them	Virtual counter displaying the number of current online users
Cooperation	Leverages drive to cooperate to complete a task or behaviour	Peer forum allows the user to post helpful tips or strategies to share with others
Competition	Leverages drive to compete against others in completing a task or action	User can contest an artificial character for a higher score during an online quiz
Recognition	Offers public recognition for a user or group	User can share their program status with friends, family or other users

PSD: persuasive systems design
