# Supplementary material for: Design and Delivery Features That May Improve the Use of Internet-Based Cognitive Behavioral Therapy for Children and Adolescents With Anxiety: A Realist Literature Synthesis With a Persuasive Systems Design Perspective
Source: J Med Internet Res. 2019 Feb 5;21(2):e11128. doi: 10.2196/11128 (PMC6379818; doi:10.2196/11128)
Supplement: Multimedia Appendix 4 [file jmir_v21i2e11128_app4.pdf]

Multimedia Appendix 4. The level of contribution and methodological quality of documents for the included internet-based cognitive behavioral therapy programs.

Document	Document type and study design[a]	Relevance			Rigor
		Level of Contribution			MMAT Score
		Context	Mechanism	Outcome	
**Program 1: BRAVE-Online for children and adolescents**					
[49]	Published study; Quantitative randomized	High	Medium	Medium	100%
[76]	Published study; Quantitative descriptive	High	High	Medium	100%
	Published study; Quantitative descriptive	High	High	Medium	100%
[77]	Thesis; Quantitative randomized	High	Medium	Medium	100%
	Thesis; Quantitative randomized	High	High	High	100%
[78]	Published study; Quantitative randomized	High	Medium	Medium	100%
[80]	Published study; Quantitative randomized	High	Medium	Medium	100%
[58]	Registered protocol; Quantitative non-randomized	Medium	Low	N/A	N/A
[59]	Registered protocol; Quantitative randomized	Medium	Low	N/A	N/A
[75]	Registered protocol; Quantitative randomized	Medium	Low	N/A	N/A
[81]	Published study; Quantitative non-randomized	Medium	Medium	High	100%
[23]	Published study; Quantitative randomized	High	High	High	50%
[82]	Published study; Quantitative randomized	High	High	High	75%
[60]	Registered protocol; Quantitative randomized	Low	Low	N/A	N/A
[79]	Published study; Quantitative non-randomized	High	High	High	75%
[61]	Program website; N/A	Low	Low	N/A	N/A
**Program 2: iCBT for children and adolescents with dental anxiety**					
[62]	Registered protocol; Quantitative randomized	Medium	Low	N/A	N/A
[48]	Thesis; Quantitative non-randomized	Medium	High	High	75%
**Program 3: Internet-delivered CBT for children with anxiety disorders**					
[63]	Registered protocol; Quantitative non-randomized	Low	Low	N/A	N/A
[64]	Registered protocol; Quantitative non-randomized	Low	Low	N/A	N/A

Document	Document type and study design[a]	Relevance			Rigor
		Level of Contribution			MMAT Score
		Context	Mechanism	Outcome	
[65]	Registered protocol; Quantitative randomized	Low	Low	N/A	N/A
[54]	Thesis; Quantitative randomized	High	Medium	Medium	75%
	Thesis; Quantitative non-randomized	High	Medium	High	100%
[66]	Registered protocol; Quantitative non-randomized	Low	None	N/A	N/A
[83]	Published study; Quantitative randomized	High	Medium	Medium	75%
[84]	Published study; Quantitative non-randomized	High	High	High	100%
[85]	Published study; Quantitative non-randomized	High	High	High	100%
[67]	Program website; N/A	Low	Low	N/A	N/A
**Program 4: Internet-delivered CBT for children with specific phobia**					
[50]	Published study; Quantitative non-randomized	High	Medium	Medium	100%
**Program 5: SmartCAT App for children with anxiety disorders**					
[68]	Registered protocol; Quantitative non-randomized	Low	Low	N/A	N/A
[46]	Published study; Quantitative non-randomized	High	Medium	Medium	100%
[69]	Program website; N/A	Low	Low	N/A	N/A
**Program 6: Internet cognitive-behavioral skills-based program**					
[51]	Thesis; Quantitative randomized	High	High	High	100%
[70]	Program website; N/A	Low	Low	N/A	N/A
[71]	Study flyer; N/A	Low	Low	N/A	N/A
**Program 7: REACH for Success App**					
[47]	Thesis; Quantitative descriptive	High	High	High	75%
	Thesis; Quantitative descriptive	High	High	High	75%
**Program 8: Individually tailored iCBT for adolescents**					
[55]	Published study; Quantitative randomized	High	Medium	Medium	100%
[86]	Thesis; Quantitative randomized	High	Medium	Low	100%

Document	Document type and study design[a]	Relevance			Rigor
		Level of Contribution			MMAT Score
		Context	Mechanism	Outcome	
**Program 9: The e-couch Anxiety and Worry Program**					
[72]	Registered protocol; Quantitative randomized	Medium	Low	N/A	N/A
[52]	Published protocol; Quantitative randomized	High	High	N/A	N/A
[87]	Published study; Quantitative randomized	High	High	High	50%
[24]	Published study; Quantitative randomized	High	High	Medium	50%
[73]	Program website; N/A	Low	Low	N/A	N/A
**Program 10: MoodGYM**					
[53]	Published study; Quantitative randomized	High	Medium	Medium	100%
[25]	Published study; Quantitative non-randomized	High	Medium	High	100%
[26]	Published study; Quantitative randomized	Medium	High	High	100%
[74]	Program website; N/A	None	None	N/A	N/A

iCBT: internet-based cognitive behavioral therapy
MMAT: Mixed Methods Appraisal Tool
N/A: Not applicable
[a]Study designs according to MMAT classifications
